# Immune microenvironments differ in immune characteristics and outcome of glioblastoma multiforme

**DOI:** 10.1002/cam4.2192

**Published:** 2019-04-30

**Authors:** Botao Zhang, Rongfang Shen, Shujun Cheng, Lin Feng

**Affiliations:** ^1^ State Key Laboratory of Molecular Oncology, Department of Etiology and Carcinogenesis National Cancer Center/National Clinical Research Center for Cancer/Cancer Hospital, Chinese Academy of Medical Sciences and Peking Union Medical College Beijing China

**Keywords:** glioblastoma multiforme, immune microenvironment, immunotherapy, interaction changes, prognosis

## Abstract

Understanding the interactions between tumors and the host immune system holds great promise to uncover biomarkers for targeted therapies, predict the prognosis of patients and guide clinical treatment. However, the immune signatures of glioblastoma multiforme (GBM) remain largely unstudied in terms of systematic analyses. We aimed to classify GBM samples according to immune‐related genes and complement the existing immunotherapy system knowledge. In this study, we designed a strategy to identify 3 immune subtypes representing 3 different immune microenvironments (M1‐M3) and associated with prognosis. The 3 subtypes were significantly different in terms of specific immune characteristics (immune cell subpopulations, immune responses, immune cells, and checkpoint gene interactions). In additional, copy number variations and methylation changes were identified that correlated with genes related to a worse prognosis subtype in the microenvironment. More importantly, in M3 (worst prognosis subtype) and M2 (best prognosis subtype), the interaction between immune cells and checkpoint genes was different, which had an important effect on the prognosis. Finally, we used risk scores of immune cells and checkpoint genes to evaluate the prognosis of GBM patients and validated the results with 3 independent datasets. Disordered interactions between immune cells and checkpoint genes result in a change in the immune microenvironment and affects the prognosis of patients. We propose that a better understanding of the immune microenvironment of advanced cancers may provide new insights into immunotherapy.

## 1 INTRODUCTION

Glioblastoma multiforme (GBM), the most malignant type of central nervous system tumor, is the most aggressive and lethal form of cancer, with an average survival time of 15 months after diagnosis.^1^, ^2^ For GBM cases in China, the 1‐ and 5‐year overall survival (OS) rates are 61% and 9%, respectively.^3^ Despite the availability of a variety of treatments, such as surgery, chemotherapy, radiation and biotherapy, death inevitably occurs from either recurrent or progressive disease; thus, scientific and clinical advances are desperately needed. In current academic research circles, there are intensive efforts to elucidate the relationship between tumors and the immune microenvironment.^4^, ^5^ Immunotherapy has attracted increasing attention, remarkable success has been achieved in cancer immunotherapies treating advanced tumors, and targeted therapy will become an important means of tumor treatment in the future.^6^ However, immunotherapy is only applicable to a substantial fraction of patients, while others either are not suitable candidates or fail to respond. This has become a difficult problem, so new standards are urgently needed to guide individualized treatment.

An imbalance between the host microenvironment and tumor cells can result in tumor invasion, progression, and metastasis. Immune responses play critical roles in carcinogenesis and the progression of solid tumors.^7^, ^8^ It has been reported that nascent transformed cells can be initially eliminated by the host immune system based on both innate and adaptive immunity.^9^ The immune environment of a primary tumor is associated with the clinical response and benefit of immunotherapy.^10^, ^11^ For example, the immune checkpoint molecules programmed cell death protein 1 (PD1) and PD1 ligand 1 (PDL1) are targets of drugs in clinical practice.^12^ Some studies have demonstrated that tumor‐infiltrating CD8 + T lymphocyte cells and intratumoral TH1‐type molecules are associated with positive responses to therapeutics by blockading PD1 and PDL1. However, therapies with an antibody targeting PD1 (anti‐PD1) displayed response rates from 17% to 21%, with some responses being remarkably durable.^13^, ^14^ The reason for this phenomenon may be tumor mutational burden^15^ or cytokine release syndrome.^16^ However immune responses and diseases are not caused by a single immune cell or checkpoint, their interaction relationship changes.^17^ Therefore, studies of the relationship between the tumor and host microenvironment have revealed that interactions between the immune microenvironment and molecules affect the therapeutic outcome of patients. Researchers have also explored promising candidate biomarkers for predictive and prognostic value to further guide the individualized treatment of patients.

This study aimed to investigate the overall immune landscape and its clinical relevance in GBMs. From The Cancer Genome Atlas (TCGA) GBM cohort, we identified 3 immune subtypes (M1‐M3) of GBMs based on the expression of global immune genes and the subtypes were related to patients' survival. Each subtype was characterized by a different subpopulation of immune cells and immune responses. The abnormally expressed genes in M3 (the worst prognosis subtype) also exhibited different methylation and copy number variation (CNV) levels from the M1 and M2 subtypes. In M2 (the best prognosis subtype) and M3, the interactions between immune cells and checkpoint genes differed significantly, and we identified 3 checkpoints (CD27, PDL1, and CTLA4) with the most significant differences between the 2 subtypes. Then, we found that the total risk scores for these 3 checkpoints and 24 immune cells were related to patients' survival, furthermore, high risk scores predicted worse prognosis. In addition, we validated these findings in 3 independent datasets. We observed that the immune microenvironment of different patients was different, which would affect the immune treatment outcome of patients. Through this method, we hope to find target markers in different immune microenvironments that interact with immune cells to predict patients' prognosis in order to guide individualized treatment.

## 2 MATERIALS AND METHODS

### 2.1 Patients and samples

The GBM samples used in this study were obtained from Beijing Tiantan Hospital between January 2005 and December 2009 (detailed information about the tissue samples is presented in Table S1). Overall survival (OS) was calculated from the date of diagnosis until death or the end of follow‐up, while progression‐free survival was defined as the time between diagnosis and the first unequivocal clinical or radiological sign of disease progression. All samples were rinsed with normal saline after surgical resection and divided into 2 portions: one was placed in liquid nitrogen immediately and then stored at −80°C until use, and the other was placed in formaldehyde solution and hematoxylin‐eosin stained to assess the percentage of tumor cells. All patients signed informed consent forms. The use of human tissue samples and the experimental procedures for this study were reviewed and approved by the Ethics Committee of the Cancer Institute and Hospital, Chinese Academy of Medical Sciences.

### 2.2 RNA isolation and gene expression microarray

Total RNA from frozen samples was extracted using the mirVana miRNA Isolation kit (Ambion 1561) according to the manufacturer's protocol. RNA concentrations were determined by an ND‐1000 UV‐VIS Spectrophotometer (NanoDrop Technologies, Wilmington, DE), and RNA integrity was evaluated using the RNA 6000 LabChip kit in combination with the Agilent 2100 Bioanalyzer (Agilent Technologies, Santa Clara, CA). The RNA samples used in this study all exhibited RNA concentrations >40 ng/μL and RNA integrity numbers >7.0. After RNA concentration and integrity analysis, the samples were analyzed using Agilent 4 × 44K Whole Human Genome Oligo Microarrays at the Cancer Hospital, Chinese Academy of Medical Sciences, according to the manufacturer's specifications. In brief, 500 ng of purified total RNA was reversed transcribed in vitro using the Low RNA Input Linear Amplification Kit PLUS (Agilent) and then transcribed into cRNA labeled with Cy3. In total, 1.65 μg of cRNA was hybridized to each microarray. After hybridization, the slides were washed and scanned with an Agilent G2505B Microarray Scanner System. The fluorescence intensities of the scanned images were extracted and preprocessed using Agilent Feature Extraction Software (v9.1). The raw data were normalized using the GeneSpring GX software program, version 11.5 (Silicon Genetics, Redwood City, CA). The raw and processed data are publicly available at the Gene Expression Omnibus (GEO) website under the accession number GSE122586.

### 2.3 Public clinical and molecular data collection

In TCGA datasets, RNAseq data (level 3, RSEM‐normalized data), methylation array data (Illumina Human Methylation 450) and CNV data (Affymetrix SNP 6.0) of GBMs were downloaded from the NIH National Cancer Institute GDC Data Portal (https://portal.gdc.cancer.gov/). Two external independent transcriptome datasets of GBMs were used for validation, namely, the Chinese Glioma Genome Atlas RNA sequencing dataset (CGGA data) and GEO microarray datasets GSE16011, as well as their corresponding clinical information.

A total of 404 immuno‐related human genes, including 4 immune response types, 24 immune cells, and 22 immune response categories, that were curated from the nCounter® PanCancer Immune Profiling Panel (NanoString) were implemented as candidate genes in this study. Detailed annotations for these 404 genes are listed in Table S2.

### 2.4 Identification of immune subtypes of GBMS by immune genes

The expression of immune‐related genes was used to identify immune subtypes. Through T‐Distributed Stochastic Neighbor Embedding (t‐SNE), the TCGA GBM cohort was divided into 3 immune subtypes, representing 3 different immune microenvironments. The optimal cluster number was determined by NbClust, an R package that determines the best number of clusters in a dataset.

### 2.5 Immune cell subpopulations and responses in GBM subtypes

We used gene set enrichment analysis to identify immune cell types that were enriched in each immune subtype. The expression level of each gene was log2‐transformed for subsequent analysis. For each patient, genes were ranked in descending order according to their expression values, and the association was represented by a normalized enrichment score. An immune cell type was considered enriched in a patient when the *P*‐value was <0.1. The percentage of each immune cell with significant enrichment in each immune subtype was calculated to compare the proportion of immune cell types in different classifications.^4^

The enrichment scores of immune responses in each immune subtype were determined by single sample enrichment analysis in the R package GSVA. Gene set variation analysis (GSVA) is used to estimate the variation in gene set enrichment through samples of expression datasets, so the enrichment scores of immune responses in each subtype to be computed can be compared.^18^

### 2.6 Regulation of abnormally expressed genes in the M3 subtype

For each subtype, the featured genes were identified by comparing the samples in this subtype with the remaining samples using Student's *t* test. Through comparing the differentially expressed genes of M3 subtype with M1 subtype and M2 subtype, respectively, we selected significantly differentially expressed genes in the M3 subtype, and then to explore the mechanisms of changes differentially expressed genes of M3 subtype via epigenetic variations and CNVs.

As for mRNA expression, median expression levels (used to summarize expression in each subtype) were computed using only samples with nonmissing values.

To study the relationship between the expression and DNA methylation of those genes, we mapped DNA methylation probes to the genes. The methylation level of a particular gene was defined as the mean value of all probes mapping to that gene. For a given gene, the beta value was evaluated within each immune subtype. In addition, we used Student's *t* test to examine whether these genes were differentially methylated in M3 subtype compared with M1 subtype and M2 subtype.

We used the GISTIC2 method to estimate the thresholded gene‐level CNV of GBMs to examine CNV changes in M3 abnormal genes across subtypes.^19^

### 2.7 Reconstruction of the immune response interaction network 

Based on the gene expression data, we reconstructed immune cell‐gene (immune checkpoint inhibitors and co‐inhibitory and co‐stimulatory markers of the innate and adaptive immune systems) networks; these immune checkpoint genes were selected from a previous report.^20^ Through GSVA to estimate the enrichment scores of each immune cells, this score can represent the expression or infiltration of immune cells. Then we used Pearson correlation to computer the correlation of the enrichment score of immune cells and the expression level of the target checkpoint genes. The network was visualized using Cytoscape.^21^ The edge weights of the network were based on the Pearson correlation coefficient between the immune cells and the target checkpoint genes. The cyan elliptical nodes represent immune cells, and the salmon octagonal nodes represent immune checkpoint genes.

### 2.8 Prognostic analysis

To identify the infiltration of immune cells and checkpoint genes that could predict GBM patient prognosis, a risk factor score was calculated to assess the survival of patients. In brief, we used univariate Cox regression analysis to evaluate the association between survival time and the infiltration of each immune cell type and the expression levels of checkpoint genes. Regression coefficients with a plus sign indicated that increased expression was associated with decreased survival, that is risky factors; conversely, a minus sign indicated that increased expression was associated with increased survival, that is protective factors. A mathematical formula was constructed to predict survival, and we assigned a risk score to each patient by the regression coefficients from the univariate Cox regression analysis.^22^ The risk score of each patient was calculated as follows:

$$\begin{array}{c} Risk\_Score=\sum_{i=1}^{n}\alpha_{i}*expression\;\left(checkpoint\;genes\right)\\ \;\;\;\;\;\;+\beta *infiltration\;(immune\;cells) \end{array}$$

α *_i_* and *β* represent the regression coefficients of gene expression values and the infiltration degree of immune cells, respectively. All patients in the dataset were thus assigned to high‐risk and low‐risk groups using the median risk score as the cut‐off point. Patients with higher risk scores were expected to have poor survival outcomes. The Kaplan‐Meier method was used to estimate the OS time for the two subgroups, and differences in survival time were analyzed using the log rank test (R package “survival”).

### 2.9 Statistical analysis

All statistical analyses in this study were performed using R software (http://www.r-project.org). T‐SNE was implemented in the R package Rtsne. Heatmaps and Circos plots were generated by the R packages pheatmap and OmicCircos, respectively. All statistical tests were 2‐sided, and a *P* value less than 0.05 was considered statistically significant.

## 3 RESULTS

### 3.1 Immune subtypes in cancer

**Figure 1:**
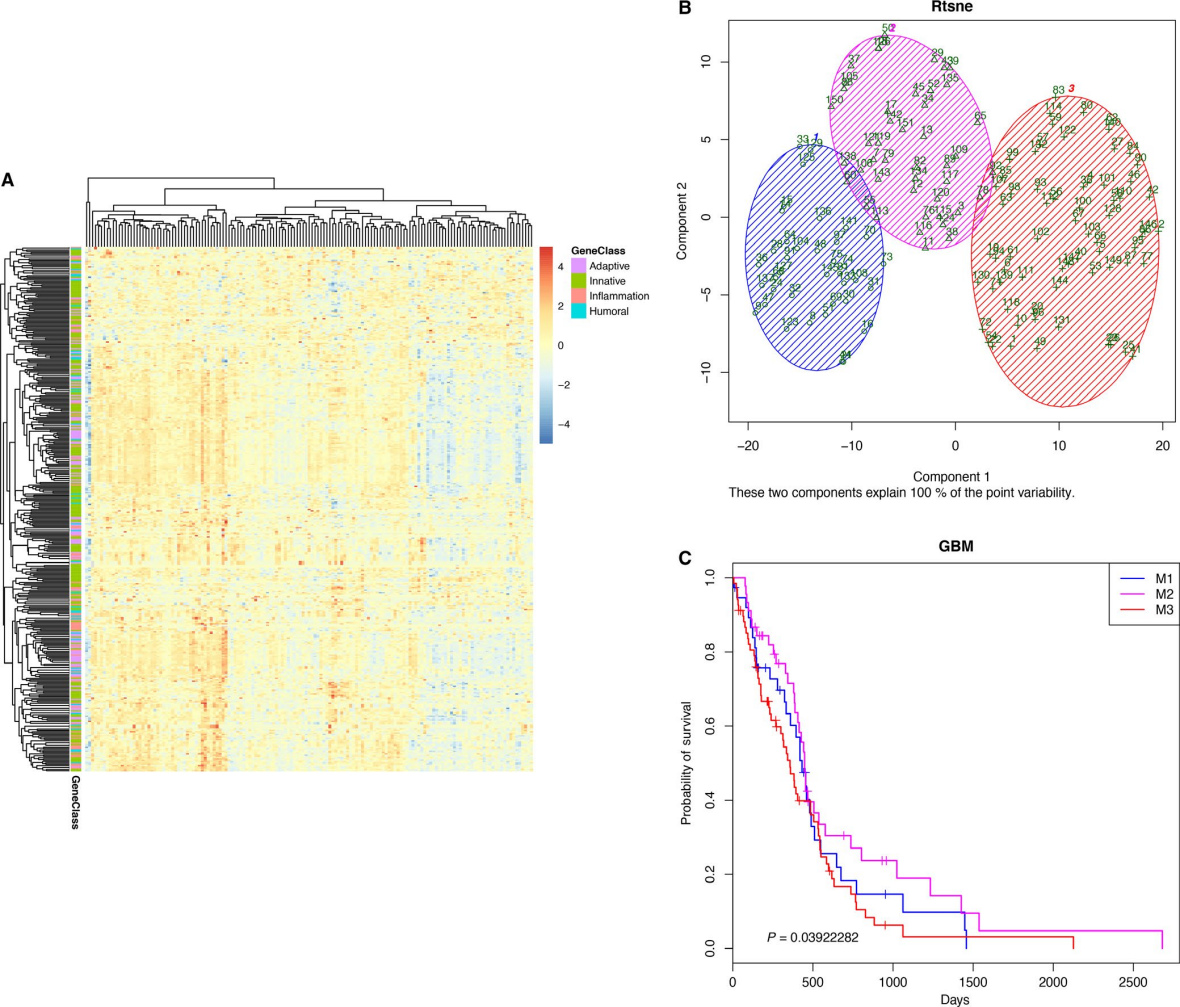
Differentially expressed immune signatures in GBM. A, The expression of an immune signature containing 404 immune‐related genes in GBM samples is shown as heatmap. Generally, these genes were classified into 4 groups: adaptive, inflammation, humoral, and innate immune response‐related genes. B, GBM samples were divided into 3 groups. The blue shading represents immune microenvironment 1 (M1, 38 samples), the magenta shading represents immune microenvironment 2 (M2, 45 samples), and the red shading represents immune microenvironment 3 (M3, 68 samples). C, Kaplan‐Meier survival plots of each subtype. The blue line represents the M1 subtype, the magenta line represents the M2 subtype, and the red line represents the M3 subtype

To characterize the immune characteristics of all TCGA GBM samples, immune signatures according to the nCounter® PanCancer Immune Profiling Panel classification into 4 immune response types (adaptive response, inflammation response, humoral response and innate response) containing a panel of 404 immune‐related genes were used to describe the immune landscape in GBM samples (Figure 1A). We observed that different immune responses in different samples were associated with differences in the immune microenvironment, additionally, the interactions between tumors and hosts were diverse. To identify common immune subtypes and evaluate whether tumor microenvironment features can predict outcomes, we analyzed the microenvironments across the landscape of all GBM samples. Four immune response types, that is 404 genes were used to perform a cluster analysis, and 3 immune subtypes were obtained, representing 3 different immune microenvironments (referred to as M1, M2, and M3, Figure 1B). Furthermore, the immune microenvironment subtypes were associated with OS. M2 had the best prognosis, while M3 had the least favorable outcome (Figure 1C). These findings suggested that the immune microenvironment affects the prognosis of patients. Therefore, we adopted the perspective of immune microenvironments to explore immunotherapy effects, which provided us with fresh clues.

### 3.2 Composition of the 3 immune subtypes

**Figure 2:**
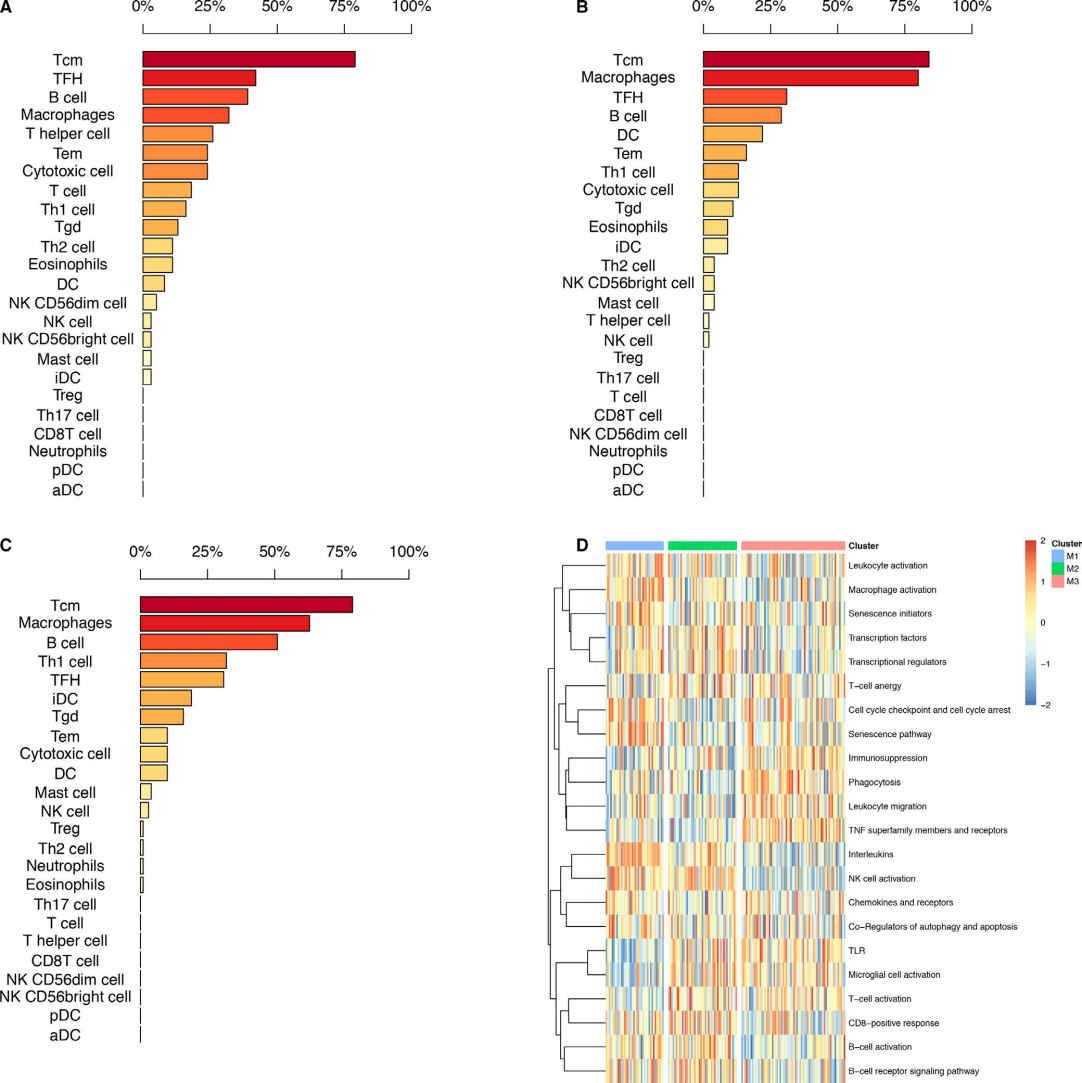
Immune cell subpopulations and immune responses in 3 immune subtypes. A‐C, Proportion of immune cell subpopulations in each immune subtype. D, Different immune responses in 3 immune subtypes

The immune cell proportions of the tumor stromal fraction varied across immune subtypes. Using a transcriptome expression dataset, we performed GSVA of 24 immune cell subpopulations, including 11 innate and 13 adaptive immune cell subpopulations. The results showed that the proportion of subpopulations of immune cells was different in each immune subtype. In M1, the immune cell subpopulations with the top 3 highest enrichments included Tcm (central memory T) cells (79%), TFH (T follicular helper) cells (42%), and B cells (39%). In M2, the top 3 highest enrichments included Tcm cells (84%), macrophages (80%), and TFH cells (31%); however, in M3, the top 3 highest enrichments were Tcm cells (79%), macrophages (63%), and B cells (51%). These results, which are shown in Figure 2A‐C, indicated that the proportion of immune cells was associated with the immune microenvironment, and different immune microenvironments affected the prognosis of patients.

In addition to immune cell subpopulation differences, through GSVA, we observed that the 3 subtypes demonstrated different immune responses. The M3 subtype (with the worst prognosis) showed high enrichment scores in immunosuppression, phagocytosis, leukocyte migration, TNF superfamily members and receptors, while the M2 subtype (with the best prognosis) exhibited high enrichment scores in CD8‐positive response, B‐cell activation, B‐cell receptor signaling pathway, transcription factors, and so on (Figure 2D).

### 3.3 Regulation of abnormally expressed genes in the M3 subtype

The abnormally expressed genes in M3 were not conducive to the prognosis of patients; therefore, we speculated that these genes might be critical for cancer occurrence and development. To further explore this theory, understanding of their expression in different states of the immune microenvironment was needed. We examined the expression and expression control of those genes via epigenetic and CNV mechanisms.

**Figure 3:**
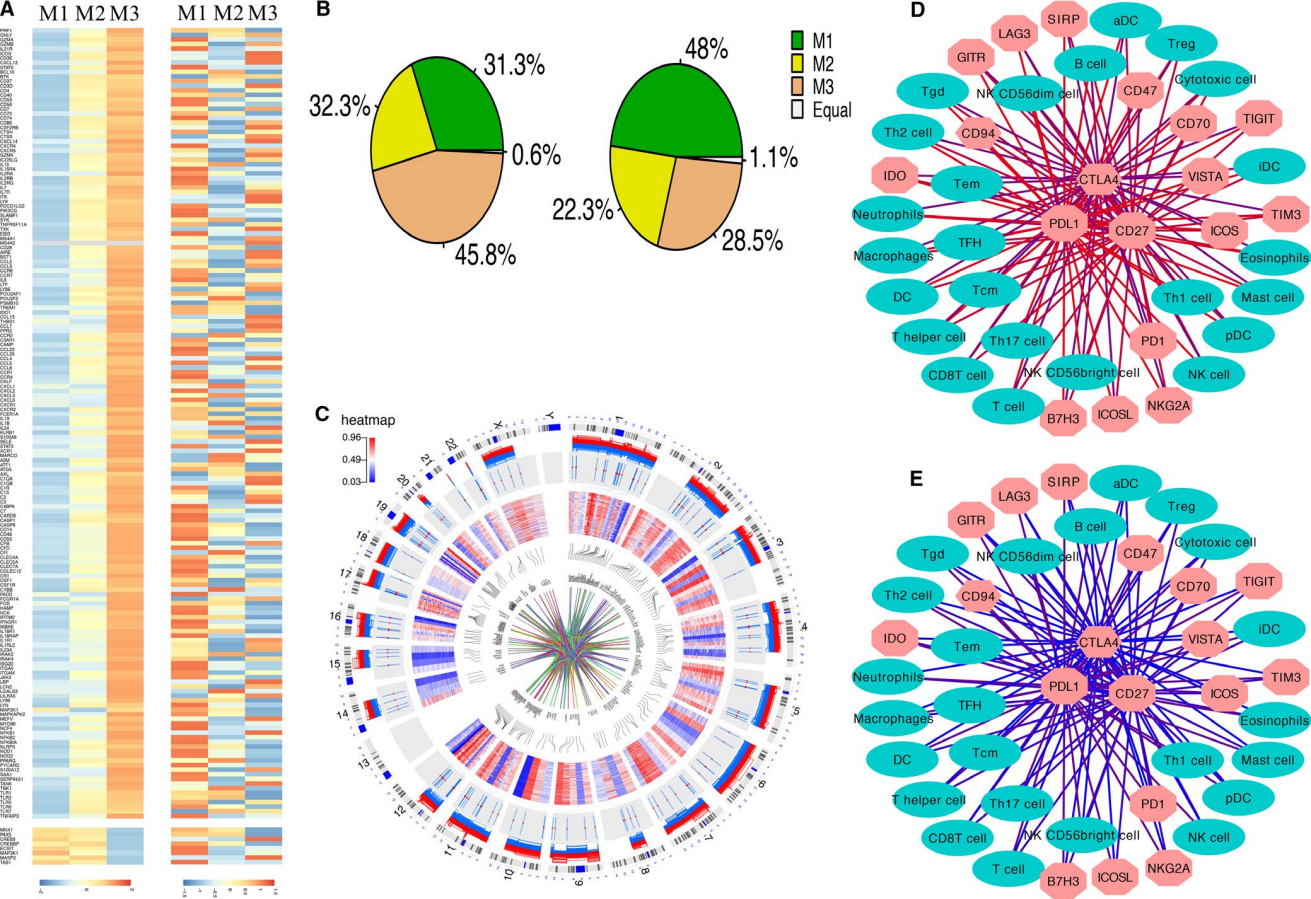
Regulation of abnormally expressed genes in the 3 subtypes and the interaction network between 24 immune cells and 18 checkpoint genes in the M2 and M3 subtypes. A, From left to right: mRNA expression (median normalized expression levels); methylation expression (median DNA methylation beta value). B, The ratio of amplification and deletion frequencies in 3 immune subtypes, respectively. C, Circos plot displaying the distribution of gene expression, DNA methylation, CNV, and interactions between genes on chromosomes. D‐E, The interaction networks between 24 immune cells and 18 checkpoint genes in the M2 and M3 subtypes, respectively. The cyan elliptical nodes represent immune cells, and the salmon octagonal nodes represent checkpoint genes. The edges represent correlation coefficients, and the colors of the edges represent the Pearson correlation coefficients, where red represents a high correlation coefficient and blue represents a low correlation coefficient

The expression profiles of 179 abnormally expressed genes in M3 immune subtype varied across different immune subtypes, perhaps indicating their role in shaping the immune microenvironment. The methylation levels of these genes in M3, there were 70 (39.1%) genes had lower methylation levels than in the M1 and M2 subtypes, and, 57 (31.8%) genes had higher methylation levels than in the M1 and M2 subtypes, indicating that the methylation levels affected the expression of multiple genes and varied across immune subtype (Figure 3A). However, the CNVs of most genes remained unchanged; yet, for the CNVs in M3 compared with the M1 and M2 immune subtypes, the amplification frequency (82 genes, 45.8%) was the highest, while the deletion frequency (51 genes, 28.5%) was higher than that of M2, but lower than that in the M1 subtype (Figure 3B). These findings suggest that changes in copy number may affect gene expression levels to some extent. The changes in the transcriptome expression, methylation and CNV levels of these genes are shown on chromosome. In the circos plot, an ideogram of a normal karyotype is shown in the outermost ring. The next outermost ring is the heatmap of gene expression at corresponding genomic coordinates, red represents high gene expression and blue represents low gene expression; the next ring represents the CNVs, multiple lines in stair steps show different individuals, red lines represent copy number amplification and blue lines represent copy number deletion; the innermost ring is heatmap illustrating DNA methylation β values, red represents high methylation β values and blue represents low methylation β values; and the middlemost lines showing the interaction between genes and genes (Figure 3C). Overall, these marked differences in M3 abnormally expressed genes may be reflective of modulation of the immune microenvironment by immune cells and cancer cells and may affect the therapeutic outcome of patients. The observed differences in the regulation of those genes might have implications for therapeutic development and combination immune therapies, and the multiple mechanisms at play in evoking them further highlight their biological importance.

### 3.4 Networks modulating immune response interactions

A number of immune checkpoint inhibitors and co‐inhibitory and co‐stimulatory markers of the innate and adaptive immune systems are currently under investigation for immunotherapy in various cancers. In an attempt to identify promising candidates for GBM immunotherapy, we reconstructed a network of immune cells and immunomodulatory molecules to explore which molecules are available. The interaction network between immune cells and these checkpoint genes was then filtered for molecules that were significantly associated with prognosis. We found that in the M2 (good prognosis) and M3 (bad prognosis) subtypes, the interactions between immune cells and checkpoint molecules were significantly different, and the interaction coefficient was higher in M2 subtype than M3 subtype, that is the stronger interaction between immune cells and checkpoint genes, the better the prognosis of patients. CD27, PDL1, and CTLA4 were at the center of the network, at the same time, the 3 checkpoint genes were the most significant changes in their interactions between M2 subtype and M3 subtype, we hypothesize that these three genes in different immune microenvironments significantly affect the prognosis of patients (Figure 3D‐E). It has been suggested that the prognosis of patients or the occurrence of disease is determined not by a single molecule but by the interaction of genes or molecules. Because different patients have significant differences in medicine sensitivity, their prognosis also differs significantly. This requires that individual treatment be adopted for different individuals in order to achieve the best therapeutic effect. Studying the interactions between target molecules and other molecules or cells and then connecting them with the prognosis of patients is of great importance for determining patients' prognosis and guidelines for clinical therapy.

### 3.5 Correlation between interaction relationship and overall survival

**Figure 4:**
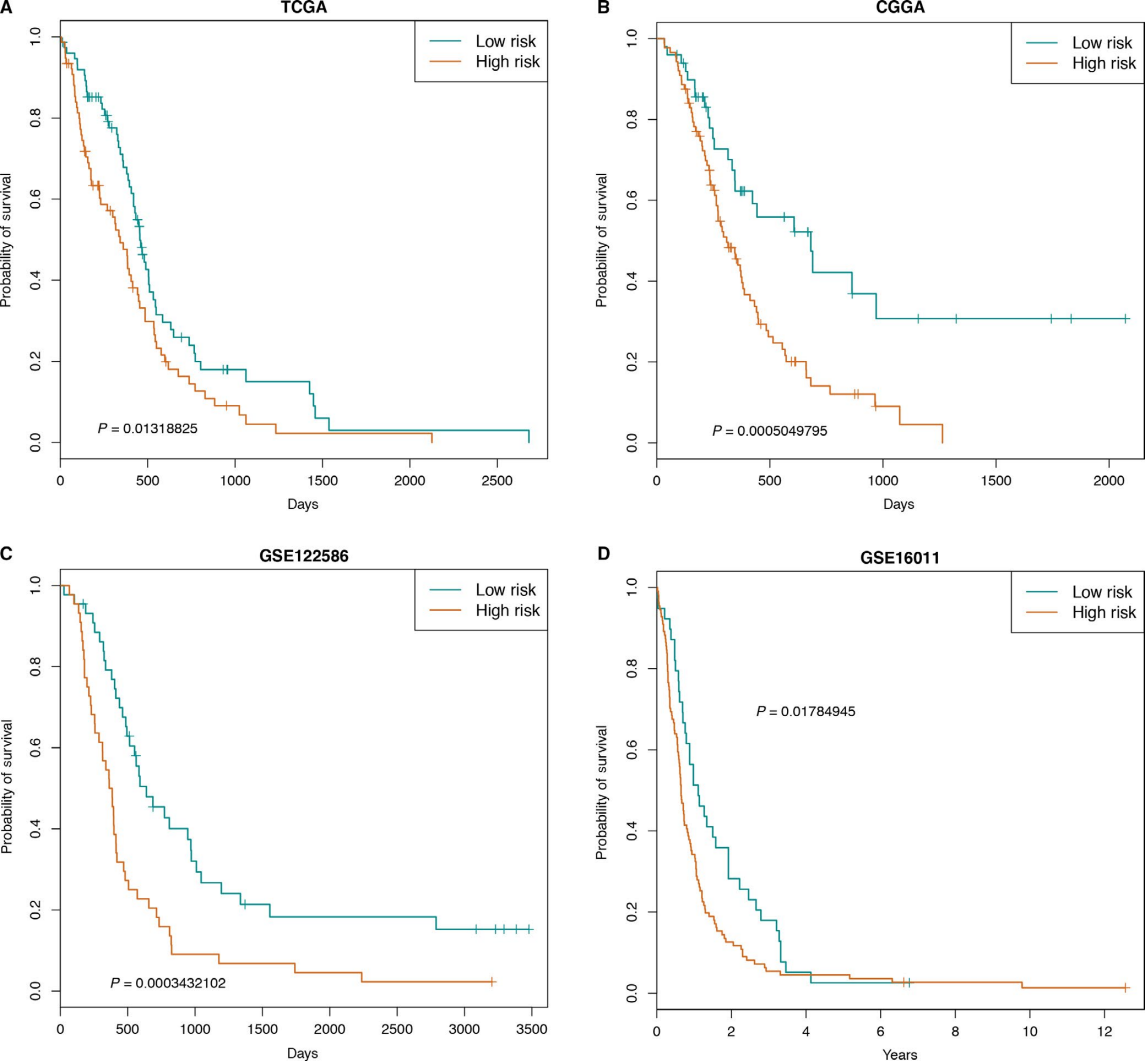
Kaplan‐Meier estimates of OS of patients according to the risk scores. A‐D, The survival curves for risk scores of 24 immune cells and 3 genes in TCGA, CGGA, GSE122586, and GSE16011 datasets

To explore the influence of network relationships on survival, we tested the prognostic value of CD27, PDL1, and CTLA4 and 24 immune cells. We performed a univariate Cox regression analysis to evaluate the association between survival time and the 3 checkpoint genes expression levels and 24 immune cells enrichment scores. In our datasets (GSE122586) and 2 external independent datasets, survival analysis indicated that the relationship could predict the prognosis of GBM patients, and high risk scores were associated with poor survival (Figure 4A‐D). These results suggested that the interaction relationship was able to predict the prognosis of GBM patients, assisting with postsurgical treatment management and providing a priori guidance for individualized treatment and targeted treatment.

## 4 DISCUSSION

We have developed an integrated strategy to characterize the interaction relationship between the immune microenvironment and the clinical outcome of human GBM. Our approach to deeply mine large datasets enabled us not only to disentangle tumor‐immune interactions but also to devise strategies for guiding cancer immunotherapy in GBM.

There is a growing body of evidence suggesting that the interaction between cancer cells and the host immune microenvironment plays a critical role in the occurrence and development of tumors.^23^ Research into immune checkpoints marks the beginning of a new era in cancer immunotherapy. However, immunotherapy works in only a small number of cancers, and only a fraction of patients have good treatment results.^24^ Further research into how to guide the clinical treatment and predict the prognosis of patients offers considerable potential.

GBM is the most malignant tumor of the nervous system, but the function of the immune response in GBM progression and prognostication remains completely unknown. High‐throughput sequencing technology provides objective data for this purpose. In this study, we divided GBM samples into 3 subtypes utilizing immune signatures. The 3 subtypes differed significantly in terms of specific immune characteristics (immune cell subpopulations, immune response, interactions between immune cells, and checkpoint genes). In additional, CNV and methylation changes were identified that correlated with genes related to a worse prognosis in the microenvironment. These findings may provide new insight into strategies for immunotherapy of GBM.

Previous studies have reported some molecular subtypes of glioma based on genome‐wide profiles.^25^, ^26^ In our analysis, we focused only on global immune‐related gene profiles, which could provide more detailed information about the immune landscape of GBM. The M2 subtype had the most favorable prognosis, suggesting that the CD8‐positive response and an increase in B‐cell infiltrates are needed for cancer control, consistent with previous reports.^27^, ^28^, ^29^ In contrast, the M3 subtype conferred the worst prognosis and displayed composite signatures reflecting immunosuppression response, leukocyte migration, and so on.

Possible impacts of methylation changes and CNVs in abnormally expressed genes in M3 were seen. Most of these genes were methylation changes, indicating that the methylation levels of these genes affected the expression profiles of multiple genes. Regarding the CNVs in M3 compared with the M1 and M2 immune subtypes, the amplification frequency was the highest, and the deletion frequency was higher than that in M2, but lower than that in the M1 subtype. These findings suggest that changes in CNV may affect gene expression levels to some extent. Further work is needed to determine the functional aspects of these associations.

The immune response is determined by the collective states of intracellular molecular networks in tumor, immune, and other stromal cells via soluble proteins such as cytokines, which mediate interactions among those cells.^30^ Therefore, studying the interactions between immune cells and molecules is important for the immunotherapy of various cancers. We found that the interactions between immune cells and immune checkpoint genes differed between the M2 and M3 subtypes. In M2 compared with the M3 subtype, the interaction coefficients between immune checkpoint genes and immune cells were greater, the disordered interactions between immune cells and checkpoint genes result in changes in the immune microenvironment and affect the prognosis of patients. Based on immune cell‐gene interactions, we identified the top 3 genes significantly different interactions with immune cells in the M2 and M3 subtypes, and then we used risk scores of 24 immune cells and 3 checkpoint genes (CD27, PDL1, and CTLA4) in 4 independent datasets to predict the prognosis of patients and to guide their clinical treatment. Of note, using these methods, it is not always possible to fully ascertain whether a particular interaction works in the tumor, immune, or stromal cell compartment, but this could be improved by incorporating additional cell type‐specific knowledge.

There are some limitations in our study. First, the histological samples were too small and not sufficiently representative to evaluate immune cell infiltration. Second, for most tumor types in TCGA, samples with fewer than 60% tumor cell nuclei according to a pathologist review were excluded from study,^31^ thus potentially excluding the most immune‐infiltrated tumors from the analysis. The degree to which this biased the results, relative to the general population of cancer patients, is difficult to ascertain. In addition, our analyses were limited by restricting them to data from molecular assays in the absence of targeted classical cellular immunology assays for confirming cell phenotype distribution, as those types of data have not been collected from TCGA patients.

In conclusion, using the gene expression profile of global immune genes, we identified 3 immune subtypes in GBM samples. These subtypes were associated with prognostic, genetic, and immune‐modulatory alterations that may shape the specific types of immune environments we observed. With our increasing understanding that the tumor immune environment plays an important role in prognosis as well as response to therapy, defining the immune subtype of a tumor may play a critical role in predicting the disease outcome as opposed to relying solely on features specific to individual cancer types. These findings regarding the intratumoral immune microenvironment may shed new light on immunotherapy strategies for advanced gliomas.

The cancer microenvironment has high prognostic value. The analysis of the immune context of tumors revealed a set of cellular and molecular immune markers that could be used to effectively and reproducibly classify patients according to their survival. Further studies are needed to validate our conclusions and to elucidate the underlying mechanisms of these phenomena.

## DISCLOSURE STATEMENT

The authors declare that they have no potential conflict of interest.

## References

[1] Binder DC , Davis AA , Wainwright DA . Immunotherapy for cancer in the central nervous system: Current and future directions. Oncoimmunology. 2016;5(2):e1082027. 27057463 10.1080/2162402X.2015.1082027 PMC4801467

[2] Nager M , Bhardwaj D , Cantí C , Medina L , Nogués P , Herreros J . Beta‐catenin signalling in glioblastoma multiforme and glioma‐initiating cells. Chemother Res Pract. 2012;2012:192362. 22400111 10.1155/2012/192362 PMC3286890

[3] Jiang T , Mao Y , Ma W , et al. CGCG clinical practice guidelines for the management of adult diffuse gliomas. Cancer Lett. 2016;375(2):263‐273. 26966000 10.1016/j.canlet.2016.01.024

[4] Angelova M , Charoentong P , Hackl H , et al. Characterization of the immunophenotypes and antigenomes of colorectal cancers reveals distinct tumor escape mechanisms and novel targets for immunotherapy. Genome Biol. 2015;16:64. 25853550 10.1186/s13059-015-0620-6 PMC4377852

[5] Li B , Severson E , Pignon J‐C , et al. Comprehensive analyses of tumor immunity: implications for cancer immunotherapy. Genome Biol. 2016;17(1):174. 27549193 10.1186/s13059-016-1028-7 PMC4993001

[6] Patel MA , Pardoll DM . Concepts of immunotherapy for glioma. J Neurooncol. 2015;123(3):323‐330. 26070552 10.1007/s11060-015-1810-5 PMC4498978

[7] Liotta LA , Kohn EC . The microenvironment of the tumour‐host interface. Nature. 2001;411(6835):375‐379. 11357145 10.1038/35077241

[8] Chew V , Han CT , Abastado JP . Immune microenvironment in tumor progression: characteristics and challenges for therapy. J Oncol. 2012;2012(5):608406. 22927846 10.1155/2012/608406 PMC3423944

[9] Kim R , Emi M , Tanabe K . Cancer immunoediting from immune surveillance to immune escape. Immunology. 2007;121(1):1‐14. 17386080 10.1111/j.1365-2567.2007.02587.x PMC2265921

[10] Hendry SA , Farnsworth RH , Solomon B , Achen MG , Stacker SA , Fox SB . The role of the tumor vasculature in the host immune response: implications for therapeutic strategies targeting the tumor microenvironment. Front Immunol. 2016;7:621. 28066431 10.3389/fimmu.2016.00621 PMC5168440

[11] Woo SR , Corrales L , Gajewski TF . Innate immune recognition of cancer. Annu Rev Immunol. 2015;33:445‐474. 25622193 10.1146/annurev-immunol-032414-112043

[12] Reck M , Rodríguez‐Abreu D , Robinson AG , et al. Pembrolizumab versus chemotherapy for PD‐L1‐positive non‐small‐cell lung cancer. N Engl J Med. 2016;375(19):1823‐1833. 27718847 10.1056/NEJMoa1606774

[13] Topalian SL , Hodi FS , Brahmer JR , et al. Safety, activity, and immune correlates of anti‐PD‐1 antibody in cancer. N Engl J Med. 2012;366(26):2443‐2454. 22658127 10.1056/NEJMoa1200690 PMC3544539

[14] Rizvi NA , Hellmann MD , Snyder A , et al. Mutational landscape determines sensitivity to PD‐1 blockade in non–small cell lung cancer. Science. 2015;348(6230):124‐128. 25765070 10.1126/science.aaa1348 PMC4993154

[15] Goodman AM , Kato S , Bazhenova L , et al. Tumor mutational burden as an independent predictor of response to immunotherapy in diverse cancers. Mol Cancer Ther. 2017;16(11): 2598‐2608. 28835386 10.1158/1535-7163.MCT-17-0386 PMC5670009

[16] Rotz SJ , Leino D , Szabo S , et al cytokine release syndrome in a patient receiving PD‐1‐directed therapy. Pediatr Blood Cancer. 2017;64(12). 28544595 10.1002/pbc.26642

[17] Hunter DJ . Gene‐environment interactions in human diseases. Nat Rev Genet. 2005;6(4):287‐298. 15803198 10.1038/nrg1578

[18] Hanzelmann S , Castelo R , Guinney J . GSVA: gene set variation analysis for microarray and RNA‐seq data. BMC Bioinformatics. 2013;14:7. 23323831 10.1186/1471-2105-14-7 PMC3618321

[19] Mermel CH , Schumacher SE , Hill B , Meyerson ML , Beroukhim R , Getz G . GISTIC2.0 facilitates sensitive and confident localization of the targets of focal somatic copy‐number alteration in human cancers. Genome Biol. 2011;12(4):R41. 21527027 10.1186/gb-2011-12-4-r41 PMC3218867

[20] Burugu S , Dancsok AR , Nielsen TO . Emerging targets in cancer immunotherapy. Semin Cancer Biol. 2017. 10.1016/j.semcancer.2017.10.001 28987965

[21] Shannon P , Markiel A , Ozier O , et al. Cytoscape: a software environment for integrated models of biomolecular interaction networks. Genome Res. 2003;13(11):2498. 14597658 10.1101/gr.1239303 PMC403769

[22] Li Y , Xu J , Chen H , et al. Comprehensive analysis of the functional microRNA‐mRNA regulatory network identifies miRNA signatures associated with glioma malignant progression. Nucleic Acids Res. 2013;41(22):e203. 24194606 10.1093/nar/gkt1054 PMC3905890

[23] Cui G , Shi Y , Cui J , Tang F , Florholmen J . Immune microenvironmental shift along human colorectal adenoma‐carcinoma sequence: is it relevant to tumor development, biomarkers and biotherapeutic targets? Scand J Gastroenterol. 2012;47(4):367‐377. 22229663 10.3109/00365521.2011.648950

[24] Yang Y . Cancer immunotherapy: harnessing the immune system to battle cancer. J Clin Invest. 2015;125(9):3335‐3337. 26325031 10.1172/JCI83871 PMC4588312

[25] Yan W , Zhang W , You G , et al. Molecular classification of gliomas based on whole genome gene expression: a systematic report of 225 samples from the Chinese Glioma Cooperative Group. Neuro‐oncology. 2012;14(12):1432. 23090983 10.1093/neuonc/nos263 PMC3499016

[26] Mur P , Mollejo M , Hernández‐Iglesias T , et al. Molecular classification defines 4 prognostically distinct glioma groups irrespective of diagnosis and grade. J Neuropathol Exp Neurol. 2015;74(3):241‐249. 25668564 10.1097/NEN.0000000000000167

[27] Matsumoto H , Thike AA , Li H , et al. Increased CD4 and CD8‐positive T cell infiltrate signifies good prognosis in a subset of triple‐negative breast cancer. Breast Cancer Res Treat. 2016;156(2):237‐247. 26960711 10.1007/s10549-016-3743-x

[28] Klebanoff CA , Gattinoni L , Restifo NP . CD8+ T‐cell memory in tumor immunology and immunotherapy. Immunol Rev. 2006;211(1):214‐224. 16824130 10.1111/j.0105-2896.2006.00391.x PMC1501075

[29] Berntsson J , Nodin B , Eberhard J , Micke P , Jirström K . Prognostic impact of tumour‐infiltrating B cells and plasma cells in colorectal cancer. Int J Cancer. 2016;139(5):1129‐1139. 27074317 10.1002/ijc.30138

[30] Vésteinn T , Gibbs DL , Brown SD , et al. The immune landscape of cancer. Immunity. 2018; 81( 1): 105.

[31] Nature. Comprehensive molecular characterization of clear cell renal cell carcinoma. 2013;499(7456):43‐49. 23792563 10.1038/nature12222 PMC3771322

